# Supplementary material for: Dynamic self-stabilization in the electronic and nanomechanical properties of an organic polymer semiconductor
Source: Nat Commun. 2022 Jun 2;13:3076. doi: 10.1038/s41467-022-30801-x (PMC9163058; doi:10.1038/s41467-022-30801-x)
Supplement: Supplementary file 1 — Supplementary Information [file 41467_2022_30801_MOESM1_ESM.pdf]

Supplementary Information

Dynamic self-stabilisation in the electronic and nanomechanical properties of an organic polymer semiconductor

Illia Dobryden[1, 2*], Vladimir V. Korolkov[3*, §], Vincent Lemaur[4], Matthew Waldrip[5], Hio-Ieng Un[6], Dimitrios Simatos[6], Leszek J. Spalek[6], Oana D. Jurchescu[5], Yoann Olivier[7], Per M. Claesson[1], Deepak Venkateshvaran[6, §]

[1]KTH Royal Institute of Technology, School of Engineering Sciences in Chemistry, Biotechnology and Health, Department of Chemistry, Division of Surface and Corrosion Science, Drottning Kristinas väg 51, Stockholm SE-100 44, Sweden

[2]Experimental Physics, Division of Materials Science, Department of Engineering Sciences and Mathematics, Luleå University of Technology, Luleå SE-971 87, Sweden

[3]Park Systems UK Limited, MediCity Nottingham, Thane Road Nottingham, NG90 6BH, United Kingdom

[4]Laboratory for Chemistry of Novel Materials, University of Mons, Place du Parc 20, B-7000 Mons, Belgium

[5]Department of Physics and Center for Functional Materials, Wake Forest University, Winston-Salem, NC 27109 USA

[6]Cavendish Laboratory, University of Cambridge, JJ Thomson Avenue, CB3 0HE, Cambridge, United Kingdom

[7]Laboratory for Computational Modelling of Functional Materials, Namur Institute of Structured Matter, Université de Namur, Rue de Bruxelles, 61, B-5000 Namur, Belgium

*These authors contributed equally to this work
§Authors to whom scientific correspondence should be addressed: dv246@cam.ac.uk and pse@parksystems.com

Sections

SI Section 1: Thin film deposition for structural analysis and nanomechanical characterisation

SI Section 2: AFM phase topography on six different regions on the spin coated C16-IDTBT film

SI Section 3: Transistor and Seebeck device fabrication

SI Section 4: Step-by-step measurement of Seebeck coefficient and transistor characteristics over time

SI Section 5: What goes into a measurement of the on-chip Seebeck coefficient?

SI Section 6: Spectral analysis of the density of trap states

SI Section 7: Molecular Mechanics and Molecular Dynamics Numerical Simulations

SI Section 8: A note on the slopes of the Seebeck coefficient with carrier density

SI Section 9: Nanomechanics of C16-IDTBT samples

SI Section 10: Time evolution of a C16-IDTBT sample with plasticizers

SI Section 1: Thin film deposition for structural analysis and nanomechanical characterisation

Deposition of C16-IDTBT films

a. We fabricated the IDTBT pristine samples from a solution concentration of 10 g/l (75% 1,2-dichlorobenzene – 25% chloroform) using the following protocol:
    i. We weighed roughly 2 mg of IDTBT powder (Mn 69 kg/mol, Mw 116 kg/mol, PDI 1.68) in air.
    ii. We purged the glovebox during the entire procedure.
    iii. We pre-heated the hot plates for 20 minutes.
    iv. We used separate glass syringes and stainless-steel needles for the two solvents.
    v. The glass syringes and steel needles were cleaned using the following protocol:
        1. The steel needle was attached to the syringe.
        2. The plunger was removed and rinsed with acetone.
        3. The syringe body was filled with the acetone and the plunger pushed the acetone through the syringe and needle into the waste beaker.
        4. The above process was repeated for IPA.
        5. The syringes and needles were blow dried with a N_2 gun.
        6. The syringes were plasma treated at 300 Watts for 10 minutes.
    vi. The glass syringes and steel needles were brought in the glovebox, wrapped in Al foil.
    vii. We rinsed the glass syringes once with their respective solvent before making the solution (i.e., the first quantity of solvent decanted was discarded).
    viii. The solution was pre-heated at 60° C for 30 minutes while purging the glovebox.
b. Once the IDTBT was completely dissolved in the solution, 17 µL of solution was sufficient for a 1 cm x 1 cm substrate. The solution was fluid enough to coat the entire substrate.
c. The IDTBT was spin coated at 1500 rpm for 60 seconds, using an initial ramp of 3s in the glovebox.
d. Samples were annealed for 1 hour at 100° C, while purging the glovebox. These processing conditions yield samples that are approximately 50 nm thick as measured using a Dektak profilometer and an atomic force microscope.
e. The samples were removed from the hotplate and stored in a DB12 plastic box.

f. For those samples that needed to be shipped to the labs of our collaborators, we thermally sealed the DB12 plastic boxes in moisture barrier ESD bags, inside the glovebox, so that they do not see air during shipment.

The samples were shipped within a couple of days to the laboratories where they were to be measured, either at KTH Stockholm or to Park Systems Nottingham, and all measurements were completed within a week to eight weeks from the date of fabrication. Multiple such samples were fabricated over several months to cross validate the measurements.

SI Section 2: AFM phase topography on six different regions on the spin coated C16-IDTBT film

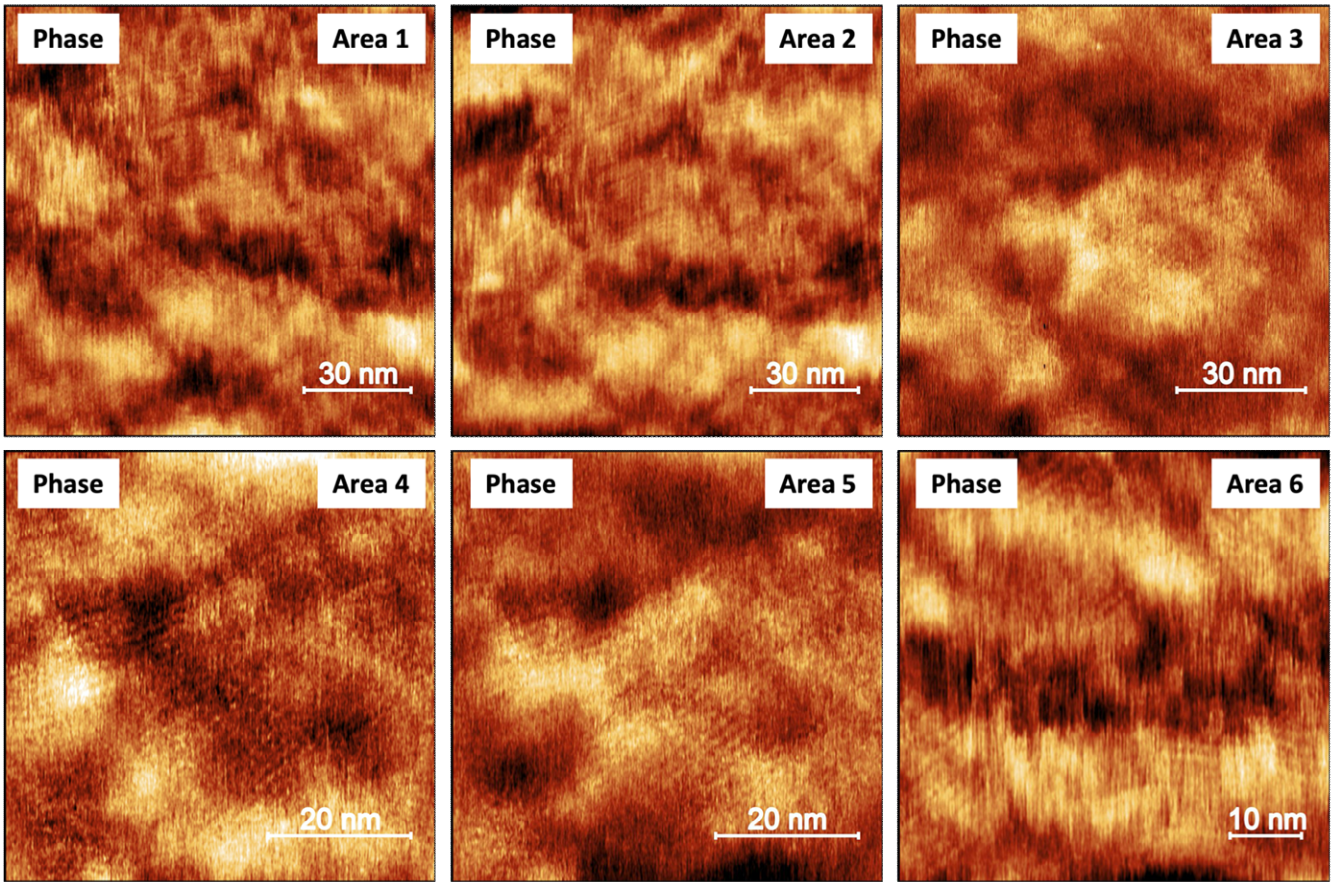

Supplementary Figure 1 Nanoscale order in spin-coated C16-IDTBT thin films. Six different regions with varying resolutions showing nanoscale order within the solution processed thin films.

SI Section 3: Transistor and Seebeck device fabrication

This section outlines the steps used to fabricate organic electronic devices for Seebeck and FET measurements. The outline presented here is written with the intention of being instructional and will hopefully prove useful to those wishing to replicate the results presented herein. The entire fabrication was done in the Microelectronics Cleanroom of the MRC building in the Cavendish Laboratory.

Substrate cleaning

A 1317F 700-micron thick Corning glass slide of 8 cm x 4 cm was diced into smaller substrates each with an area of 2 cm x 2 cm using a diamond scriber pen. The individual substrates were then sonicated in a solution containing a cap full of Decon 90 detergent and 100 ml of DI water, followed by pure DI water, Acetone and IPA each for 10 minutes. Samples were then blow dried in nitrogen, placed on a glass Petri dish and plasma ashed in a TEGAL oxygen plasma asher for 10 minutes at 250 W. After the plasma ashing step, the samples were once again sonicated in Acetone and IPA for 3 minutes each. They were then blow dried in nitrogen.

Photolithography

Photoresist LOR 5B was spin coated onto the substrates at 6000 rpm for 30 seconds from a solution filtered using a PTFE filter with 5-micron pores. The substrates with LOR 5B were baked at 190 degrees Celsius for five minutes on a hotplate. S1813 photoresist was then spin coated at 6000 rpm for 30 seconds and baked at 120 degrees Celsius for two minutes. The bilayer resist is needed to achieve an undercut necessary for high-throughput photolithography. Using a photomask, the required electrode pattern was transferred onto the resist using a Karl Suss MJB3 mask aligner with an exposure time of 10 seconds in the soft contact mode. The samples were then developed in MF319 with slight agitation for 45 seconds, rinsed in DI water for 1 minute and blow dried in nitrogen. An optional following step was to place the samples in the plasma asher for a few minutes after this to clean the exposed glass areas of any photoresist.

Fabrication of device source and drain electrodes (which also act as on-chip thermometers)

For IDTBT devices, a Cr/Au bilayer was used. 5 nm Cr acted as an adhesion layer followed by 20 nm Au. The layers were thermally evaporated in an Edwards Auto 306 four-turret evaporator. The vacuum level of the

evaporator was in the 10^{-6} mbar range. The devices with a uniform coating of the bilayer were then placed in NMP solution for a few hours for the lift-off to take place. After successful lift-off, the samples with the patterned electrodes were sonicated in Acetone and IPA for 10 minutes each, followed by blow drying them in Nitrogen.

Solution-processed organic semiconductors

The organic active layers were all processed from dissolved solutions of the organic material in a solvent. In the case of IDTBT, 1,2 Dichlorobenzene (DCB) or a mix of 1,2 DCB and Chloroform was used as the solvent. 10 mg of the organic material was dissolved in 1 ml solvent. The organic materials were dissolved in the solvent by heating the solution to 80 degrees Celsius in a nitrogen glovebox and stirring the vial manually now and then till the complete dissolution was achieved. The process takes between 30 minutes and an hour when on the hot plate. The films were produced using the deposition procedure described earlier. An optional step before the deposition of the organic was to treat the substrates/electrodes in oxygen plasma for 10 minutes at 300 W.

Device patterning process

In the case where an unpatterned device would serve the purpose, a dielectric layer was directly spin coated onto the organic active layer. The dielectric layer used with IDTBT was Cytop M-grade (CTL-809M). 500 nm Cytop was achieved by spinning it from a solution diluted with the Cytop solvent CT-180 in a volume ratio of 3:1 (i.e., 3 parts of Cytop in 1 part of the Cytop solvent). The solution was spun at 500 rpm for 3 seconds followed immediately by 2000 rpm for 60 seconds. The ramp to 500 rpm was 1 second and the ramp to 2000 rpm was 3 seconds. The Cytop layer was dried at 90 degrees Celsius for between 20 and 30 minutes. The above relevant steps were performed in the nitrogen glovebox immediately after solution processing the organic active layer and were used for devices that did not require patterning, such as when the goal is to fabricate and measure only organic transistors.

In the case of the Seebeck device requiring a patterned organic semiconductor, a sacrificial layer of Cytop from a solution of 1:2, i.e., 1-part Cytop in 2-parts Cytop solvent, was spin coated on the active organic layer. The layer was spin at 1000 rpm for 60 seconds and baked at 90 degrees Celsius for 20 minutes in the nitrogen glovebox. The device was then taken out of the nitrogen glovebox and loaded immediately into the four-turret evaporator with not more than 5 minutes of air exposure. In the evaporator, a 1 nm Al layer was evaporated onto Cytop. This

was needed to change the surface energy of Cytop and make it easier to spin coat a resist layer on it. The 1 nm Al layer was evaporated under a pressure of 10^{-3} to 10^{-4} mbar. After the deposition of this Al layer, S1813 was spin coated onto the device at 4000 rpm and baked at 80 degrees Celsius for 2 minutes. Photolithography was then done using the mask whose pattern was to be transferred onto the organic layer. The exposure was done in four steps of 3 seconds each, rather than one long exposure for 10 to 12 seconds. This was proven to minimise the number of air gaps or bubbles that were formed between the organic and the Cytop layers owing to a tension that builds in Cytop when S1813 alters upon exposure. The device was then subject to development in MF319 for 35 seconds and carefully blow dried in nitrogen to avoid accidentally delaminating Cytop. The dimensions of the patterned organic layer on the Seebeck device were typically 1 mm x 200 microns. The device was then placed on a Petri dish in the oxygen plasma asher such that the device is directly over one of the metal bars of the cage. Plasma ashing was done at 300 W in steps of 3 minutes with a gap of 5 minutes. This procedure was adopted to avoid heating the device or heating the entire asher. To ash away the sacrificial layer as well as the organic layer for the polymers, a total ashing time of around 8 to 10 minutes was required. Once the organic layer was patterned by ashing, 3M scotch tape was gently laid over the devices and the devices together with the tape were taken back into the nitrogen glovebox in which the scotch tape was pealed of. The scotch tape takes with it the thick Cytop-resist bi-layer over the patterned organic. Finally, the 500 nm organic dielectric Cytop layer was deposited on the device using the parameters outlined at the start of this section. The whole patterning process necessitated not more than 60 minutes in air, from the point of removal from the glovebox with the sacrificial Cytop layer, to the point of re-entry into the glovebox for the deposition of the actual device Cytop dielectric.

Evaporation of a gate electrode

After processing the organic semiconductor and the organic dielectric layer, a 25 nm thick Au stripe, 1.5 mm wide and several mm long, was evaporated onto the device to define the gate electrode. The evaporation was done through a shadow mask in vacuum of around 10^{-7} mbar, and the sample was left at this vacuum level for several hours before the evaporation was done. Immediately after fabrication, the device was taken into the glovebox and stored overnight.

Till date, there is no documented account of significant degradation to devices that are patterned using this procedure compared with devices that are unpatterned. All earlier publications from our group on the gate

modulated Seebeck coefficient in IDTBT were performed on such patterned devices, and the transistor characteristics that were derived from them showed ideal characteristics within a reasonable time from fabrication.

SI Section 4: Step-by-step measurement of Seebeck coefficient and transistor characteristics over time

Extracted measurement log from lab book when the measurements were performed:

06/02/2017

11:00 pm - Completed Device Fabrication (ended with gate evaporation) Device tested in a glovebox and left there overnight.

07/02/2017

11:00 am - Device loaded in Desert Cryogenics low temperature rig (low T rig). Transfer, Output and Seebeck measured in vacuum after pump down to low pressures to avoid any hysteresis stemming from presence in ambient conditions.

01:15 pm - Device taken out of low T rig and left in air for 2 and a half hours.

03:45 pm - Device once again put into the low T rig and pumped down in vacuum. Transfer, Output and Seebeck measured.

04:05 pm - Device taken out of low T rig and left in air for an hour (3 and a half hours in total)

05:15 pm - Device once again put in low T rig and pumped down in vacuum. Transfer, Output and Seebeck measured.

05:45 pm - Vacuum was broken and the device was left in air again for three hours (6 and a half hours in total)

08:30 pm - Device was put in vacuum again, and Transfer, Output and Seebeck were measured. Right after the measurements, the cryostat chamber was vented, and the device was exposed to ambient air.

08/02/2017

09:15 am - Device was put in vacuum again, and Transfer, Output and Seebeck were measured after a total of 18.5 hours of air exposure.

09:40 am - Vacuum was broken and the device was left in air again

02:40 pm - Device was put in vacuum once again, and Transfer, Output and Seebeck were measured after 23.5 hours cumulative of ambient exposure.

03:00 pm - Vacuum was broken and the device was left in air once again

09/02/2017

10:00 am - Device was put in vacuum again, and Transfer, Output and Seebeck were measured after 42.5 hours of cumulative air exposure. The device was exposed to air after the sequence of measurements.

10/02/2017

11:00 am - Device was put in vacuum again, and Transfer, Output and Seebeck were measured after 67.5 hours. The device was exposed to air after the sequence of measurements and left in air for about a month.

07/03/2017

Device was put in vacuum again, and Transfer, Output and Seebeck were measured. This last set of measurements constitutes the data shown after one month of air exposure.

A few additional details on the conditions under which the measurements were performed.

1. Both the transistor characterisation and the Seebeck measurements were performed in a reconfigured desert cryogenics probe station with optical access. The device had a few wires connected to it via an external high-vacuum feed-through, but also made use of the four available micromanipulator probe arms fitted with the BeCu 10-micron probe tips for electrical measurements. The device was designed in a fashion to have the electrical contact pads several millimetres away from the active area of the device.

2. The additional wires were necessary to make contact because the device has 9 contact pads; one gate electrode and four for each resistance thermometer which also doubled back as source and drain electrodes. The device sat on a temperature-controlled metal chuck throughout the measurements. To accommodate for the extra wiring, the inner aluminium chamber that sits within the vacuum chamber was not covered with a UV filter. The outer vacuum chamber contained the optical access window necessary for repositioning the probe tips during measurement under vacuum. What this meant is that the device was exposed to light during and intermittently when the lab lights were on, even if not continuously. A lamp light was shone in the optical access when the electrical probes needed to recontact the device. Because

of the way the experiments were setup and conducted, we must account for the presence of light during the healing/aging process.

3. The sample was pumped down to vacuum (around 10^{-5} mbar) in the desert cryogenics low temperature probe station where the transistor transfer curves and the Seebeck measurements were conducted. Measurements in vacuum were necessary to avoid hysteresis. The chamber was then vented to allow ambient air into it. A documented amount of time was allowed to pass (as detailed previously in this section) after which the chamber was pumped down once again, and the measurement cycle was repeated. For the measurements we took after a month, the device was removed from the cryostat and reinstalled at the time of final measurement. During this time the sample was left in ambient atmosphere and exposed to laboratory lighting.

4. Multiple contributions can influence the changes seen in the device and they most likely occur simultaneously, but on different time scales. The various contributions are detailed below:

    (a) There is the influence of ambient oxygen in the presence of light that we have tried to use here as justification, based on previous reports.

    (b) There is the influence of small amounts of ozone which can play a similar role as oxygen.

    (c) There could be the potential influence of water incorporation from atmospheric humidity that could enter the device over time and degrade its performance (something we don't observe).

    (d) There is the influence of solvent sweating on account of residual DCB leaving the device over time (which we have tried to measure using the adhesion channel in nanomechanics).

    (e) There could be structural reorganisation within the film upon solvent sweating. It is difficult to measure the nanoscopic order in fresh films with solvent because the surface of the film can be quite sticky initially. We can only measure the surface nanoscale order after many weeks.

For this reason, it has been difficult to claim either way that the IDTBT polymer film contains nanoscale order from the outset, or whether there is a slow ordered growth over time (the latter is a speculation that we now make based on additional trap-density of states simulations in Supplementary Information Section 6)

(f) There could be an influence of other trace gasses in the atmosphere whose direct impact is difficult to quantify.

(g) In the spirit of identifying all potential causes for the presence of trap states, we point out that the photolithographic patterning process used to fabricate the Seebeck devices (explained in detail in the Supplementary Information), might also be a cause of trace amounts of initially degraded performance, although such degradation has been strongly contested in the literature. The photolithography process was documented earlier by a former member of the Optoelectronics group in Cambridge, J. F. Chang *et al.*, Adv. Funct. Mater. **20**, 2825–2832 (2010), and showed no sign of such anticipated degradation. The understanding is that throughout the patterning process, the organic is protected by a relatively thick bilayer of cytop/photoresist, and this acts like a protective layer for the C16-IDTBT layer.

(h) The presence of any residual chlorinated solvents in the film can form water solvent azeotropes as already mentioned in our manuscript through the referenced work of M. Nikolka *et al.*, Adv.Mater. 30, 1801874 (2018). This if anything, is meant to remove the adverse effects of water incorporation in the device initially.

It is precisely for the potential multitude of reasons that can affect an organic transistor's performance initially, that we attempted to look at the temporal evolution of the transistor characteristics of devices fabricated from established procedures with documented/guaranteed high mobility. We consider all the influences mentioned above as intrinsic to the nature of the device when measured for the first time after fabrication under as clean and controlled conditions as possible within our laboratory.

SI Section 5: What goes into a measurement of the on-chip Seebeck coefficient?

Calibration of on-chip thermometers

As the first step towards measuring the Seebeck coefficient of the organic semiconductor using a microfabricated on-chip architecture, the resistance thermometers on-chip are calibrated in a four-point probe geometry as shown in Supplementary Figure 2 (a). The voltage across the heater is stepped as shown in Supplementary Figure 2 (b), and 10 data points are accumulated at each step in the heater voltage. The Labview program used to perform the calibration integrates a waiting time of up to 10 seconds to avoid any thermal transients and performs measurements at equilibrium and in the steady state. The corresponding current in the heater is shown in Supplementary Figure 2 (c) and is the cause for on-chip Joule heating. The heater is driven using one Voltage Source Unit (VSU) on an Agilent Semiconductor Parameter Analyser (SPA) HP 4155B, and the current from the heater is read by one common/grounded Source Measure Unit (SMU) on the same SPA. The corresponding heater power is shown in Supplementary Figure 2 (d). For these heater power steps, the four-point probe resistance of both the hot and the cold sensors on-chip are measured separately using a Keithley 2400 source meter and is plotted in Supplementary Figure 2 (e) as a function of the same sampling steps on the previous plots. These traces are measured at base/chuck temperatures between 240 K and 300 K, but the plot shown is for 300 K. The first 10 resistance points at each base/chuck temperature for no heater power is averaged. These average resistances are plotted in Supplementary Figure 2 (f) to extract the temperature coefficient of resistance, which is the change in on-chip resistance of the thermometer with a degree rise in temperature. The slope on Supplementary Figure 2 (f) is used as a calibration for the temperature differences in the device between the source and the drain over several tens of microns. The green bars within the data points in Supplementary Figure 2 (f) are the statistical errors on the resistance measurement that arise from averaging 10 points and are very small.

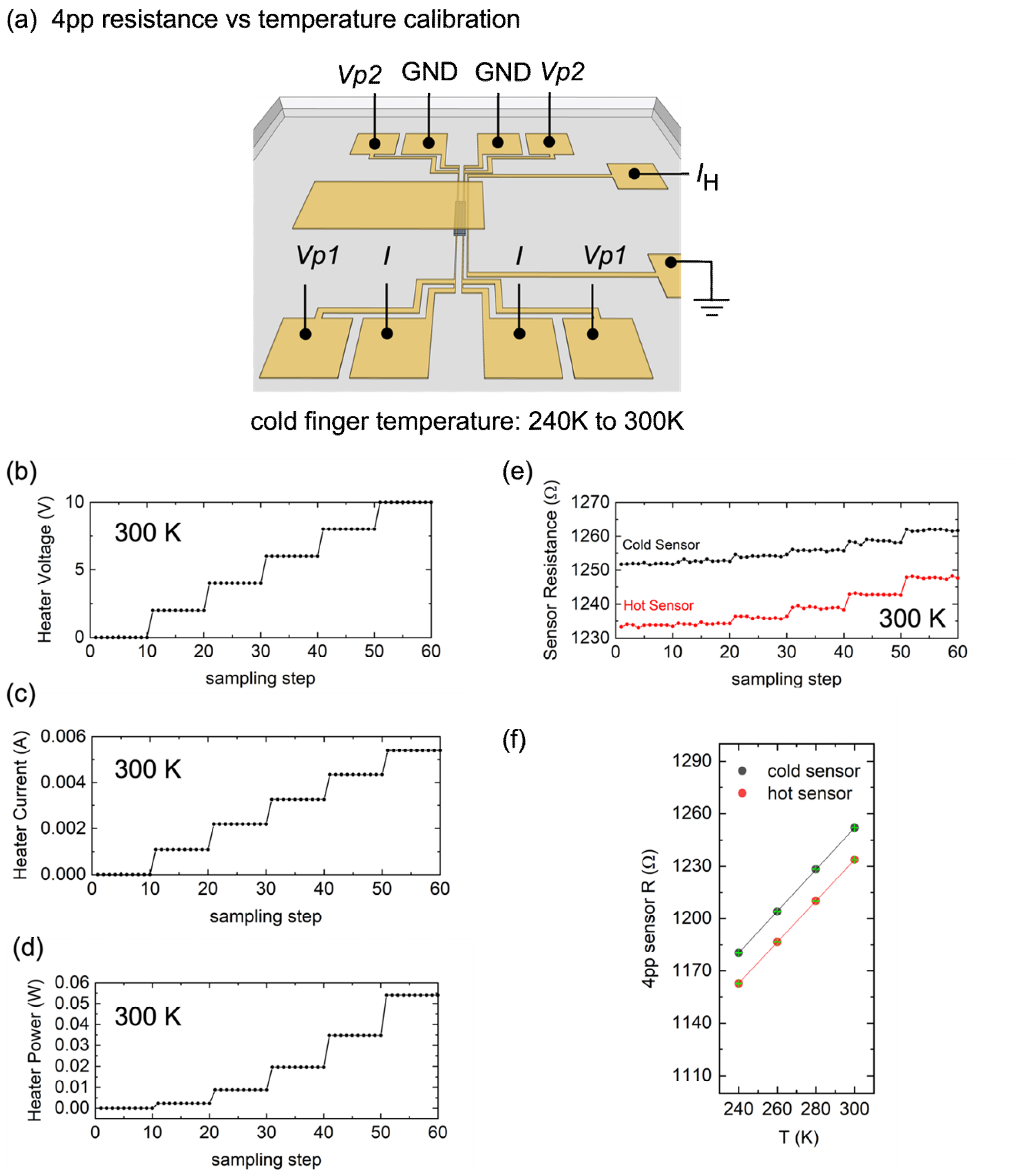

Supplementary Figure 2 Calibration of temperature coefficient of resistance of the on-chip thermometers. (a) Schematic of the device in the measurement configuration for four-point probe resistances of the hot and cold sensors. (b) On-chip applied heater voltage as a function of sampling step on the data acquisition program. (c) Corresponding on-chip measured heater current. (d) On-chip measured heater power that causes Joule heating and an on-chip temperature gradient. (e) Corresponding change in resistances of the hot and cold ends of the

device. (f) Extraction of temperature coefficient of resistance, i.e., calibration of the on-chip metal stripes to act as thermometers. Slopes of hot and cold temperature sensors are 1.179 +/-0.003 and 1.193 $+/-$ 0.005 Ω/K, respectively.

Measurement of thermal voltage

Once the temperature coefficient of resistance of the sensors on-chip is quantified, the next step in the measurement of the Seebeck coefficient is to measure the in-built thermal voltage across the organic semiconductor under the same applied heater powers. This is done by using one VSU and one grounded SMU for the heater as before, one SMU in zero current mode (high input impedance) for the thermal voltage at the cold end, and one SMU in common/ground at the hot end. The measurement probes an open circuit voltage between the source and the drain electrodes. A third SMU is used to apply a gate voltage. This measurement configuration is shown in Supplementary Figure 3 (a). The measurement is set up such that if the organic system has an excess number of holes upon accumulation, a positive thermal voltage is measured. Supplementary Figure 3 (b) show the parabolic traces in heater power that are applied for a fixed applied gate voltage. The latter is shown in Supplementary Figure 3 (c). The corresponding on-chip sensor resistances for these parabolic heater sweeps is shown in Supplementary Figure 3 (d), and their translation into temperatures using the previously determined temperature coefficient of resistances is plotted in Supplementary Figure 3 (e). At low heater powers, the error on the computation of the hot and cold sensor temperatures is on the order of the change in their temperatures, which is why only four data points at higher heater powers are selected here to compute the temperature difference between the hot and cold ends of the organic semiconductor. This temperature difference across the 50-micron device channel is shown in Supplementary Figure 3 (f). In a separate step, the thermal voltage traces are determined for the heater power sweep of Supplementary Figure 3 (b). These thermal voltages are shown in Supplementary Figure 3 (g) for the time stamps shown in the main paper, as the device heals under ambient oxygen exposure.

It is clear from Supplementary Figure 3 (g), that the offset voltage (at zero heater power) in the measured thermal voltage traces reduces as the device sits in ambient air. For each set of data at a given exposure time and for different gate voltages however, this voltage offset remains reasonably constant. Right after fabrication, the offset

is very high and negative. Under the influence of a temperature gradient and a negative gate voltage that accumulates holes, the thermal voltage gets more and more positive. This is an indication that the built-in thermal voltage itself is positive on account of hole carriers, but that it sits on a large negative voltage offset, possibly arising from an asymmetry between the potentials of the floating and the grounded SMUs. There is also a potential contribution from the device being very resistive initially, thus making an unambiguous open-circuit measurement very difficult. As the device heals in ambient air, carrier injection is improved on account of a reduction in the contact resistance, and the device becomes more conductive on account of an increased fraction of holes. These are simultaneous effects which cannot be separated and need to be treated as one influence, with importance given to the overall effect in the measurement. The initial Seebeck measurements right after fabrication were not included in the main paper since the offset voltage was very large, and on the order of the measured voltage at the highest heater powers. This may cause the extracted magnitude of the Seebeck coefficient immediately after fabrication to contain errors. Typically, the measurement offset voltages should be no larger than 10% to 20% of the maximum measured open circuit thermal voltage for reliable measurements in the corresponding author's experience. On most gated samples in the past, the offset voltage is typically between 20 microvolts and 200 microvolts on the SPA that was used, with an absolute minimum of 2 microvolts if the sample was highly conductive. The latter is the resolution limit on the SPA instrument. The fact that the offset voltage reduces from a large negative voltage to a small positive voltage over time upon air exposure in the current measurements does not seem to be a coincidence. It supports the trend of the electronic device going from showing ambipolar characteristics initially, to being unambiguously unipolar over time. The observation nevertheless demands greater investigation to understand the true origins of such an offset.

(a) Thermal voltage traces as a function of gate voltage

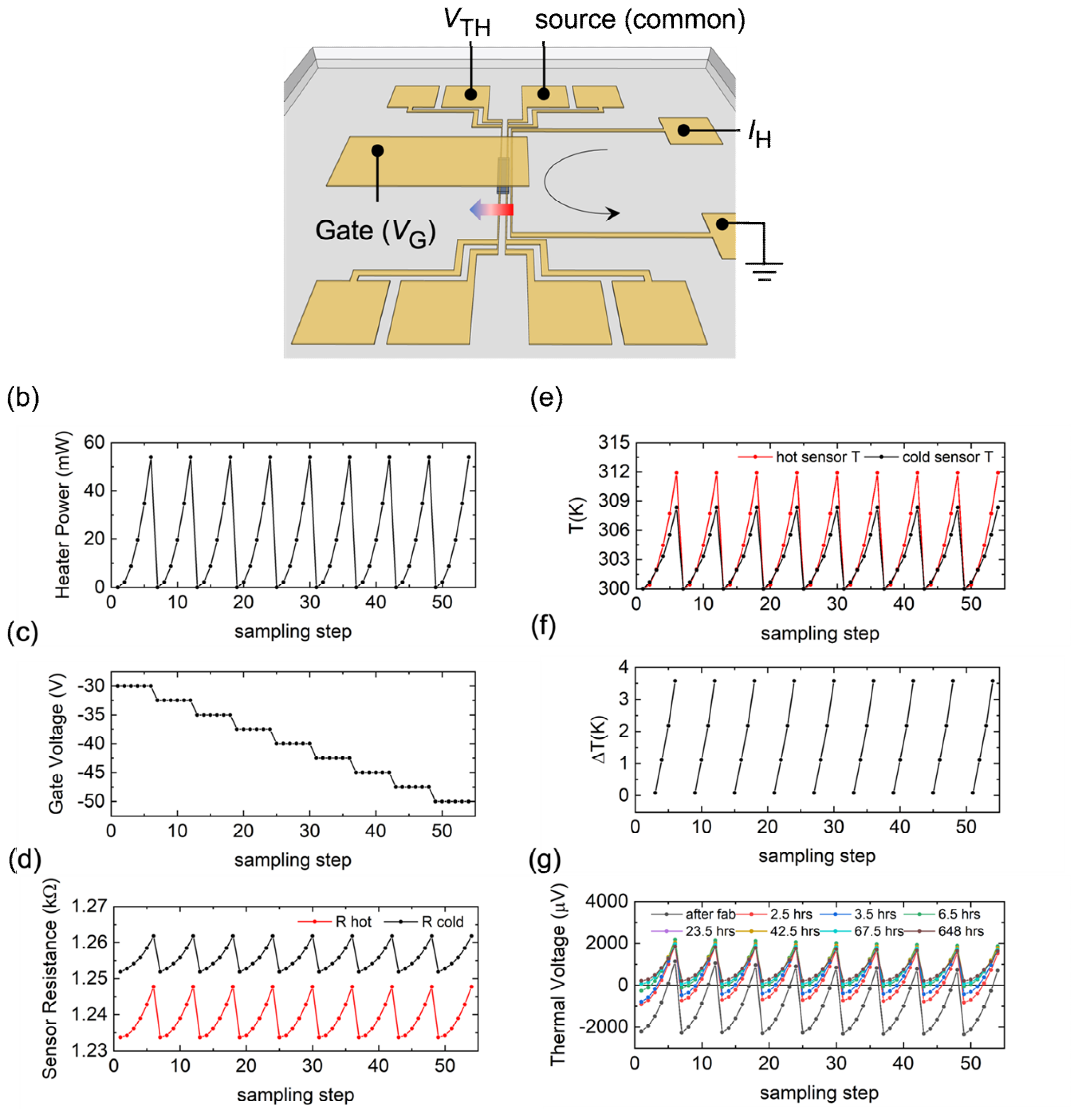

Supplementary Figure 3 Measurement of thermal voltage across the organic semiconductor. (a) Schematic of the device in the measurement configuration to acquire the thermal voltage under a temperature gradient. (b) On-chip applied heater power as a function of sampling step on the data acquisition program. (c) On-chip gate voltage in the device for every heater power. (d) On-chip change in resistance thermometers at the hot and cold ends. Each data point is an average of 10 data points for the same heater power from Supplementary Figure 2 (e). (e) Calculated temperature at the hot and cold ends. (f) Calculated temperature gradient across the channel

based on the calculated temperature at the hot and cold ends. (g) Measured open circuit thermal voltage as a function of the corresponding gate voltage sweeps, and as a function of time in ambient air. The offset voltage is constant for all gate voltages for each sweep but goes from negative to positive as the device heals. The measured hole/positive polarity thermal voltage sits on this offset voltage.

Extraction of gate voltage modulated Seebeck coefficient

Supplementary Figure 4 (a), (b), (c), (d), (e) and (f) show linear traces of the measured thermal voltage as a function of different temperature differentials, different gate voltages and at different amounts of air exposure. The Seebeck coefficient is the slope of these lines at different gate voltages. It is a linear first-order thermoelectric transport coefficient, and so effort was made to ensure linear temperature gradients between the source and drain electrodes by virtue of relatively short channel lengths in the device. The drift in the temperature of the centre of the organic semiconductor under the application of a small temperature differential was no more than a couple of Kelvin and was well below the 300 K at which all the measurements were performed.

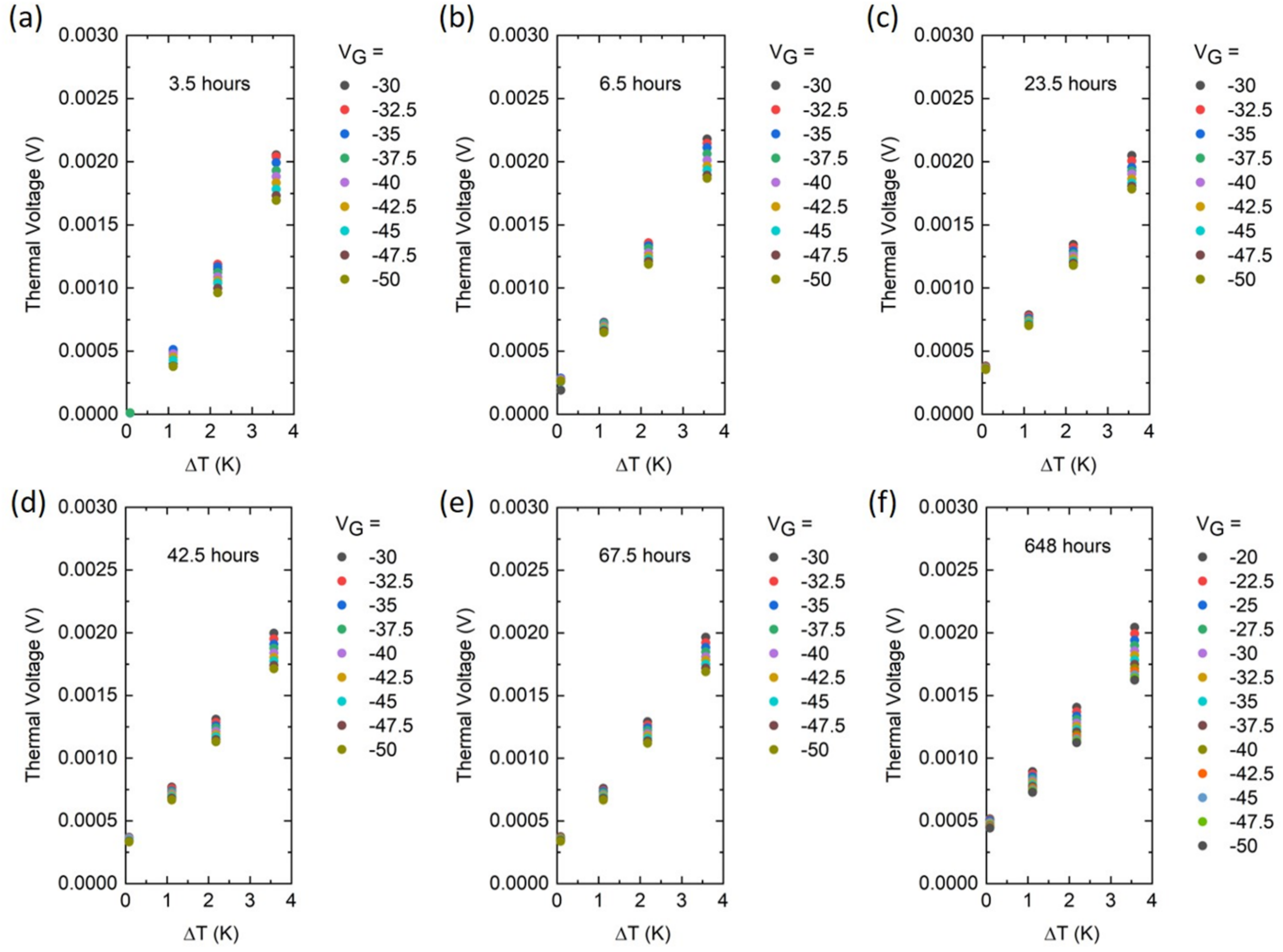

Supplementary Figure 4 Extraction of the gate modulated Seebeck coefficient in the organic semiconductor. (a), (b), (c), (d), (e) and (f) are plots of the thermal voltage as a function of the corresponding temperature differential, under different gate voltages, and for 3.5 hours, 6.5 hours, 23.5 hours, 42.5 hours, 67.5 hours, and 648 hours of air exposure respectively.

Seebeck coefficient for a particular gate voltage of V_G = -30 V and an additional note on the offset voltage

As an example of how the plot of the Seebeck vs time in air was constructed for the main paper, Supplementary Figure 5 (a) shows the built-in thermal voltage vs the applied temperature difference in the C16-IDTBT device as it undergoes improvement towards hole stability in ambient air. Supplementary Figure 5 (a) plots the linear traces at a fixed gate voltage of V_G = -30 V and for different times from the point of fabrication. As is evident, the offset voltage is initially large and negative, but gradually reduces to be small and positive over time. The Seebeck coefficient is the slope of these curves and is plotted in Supplementary Figure 5 (b). The error bars on the Seebeck coefficient come from the uncertainty in the linear fits from Supplementary Figure 5 (a). The offset voltage is not expected to influence the absolute magnitude of the Seebeck coefficient if it is not on the order of the maximum measured thermal voltage. As it may however play a role in the estimate of the Seebeck coefficient immediately after fabrication, we chose to avoid bringing it into the discussion to avoid ambiguity. The gradually reducing voltage offset in the thermal voltage measured on the SPA goes from negative to positive with time and does not appear to be a coincidence. We attempted to rationalise this sign through the presence of electrons in the channel, or an electron asymmetry between the floating SMU at the hot end and the grounded SMU at the cold end, but its exact origin remains a question. In the corresponding author's experience, and from previous published work on the gate voltage modulated Seebeck coefficient in organic semiconductors, the voltage offset was typically small, positive, and on the order of 10s and up to a few hundred microvolts.

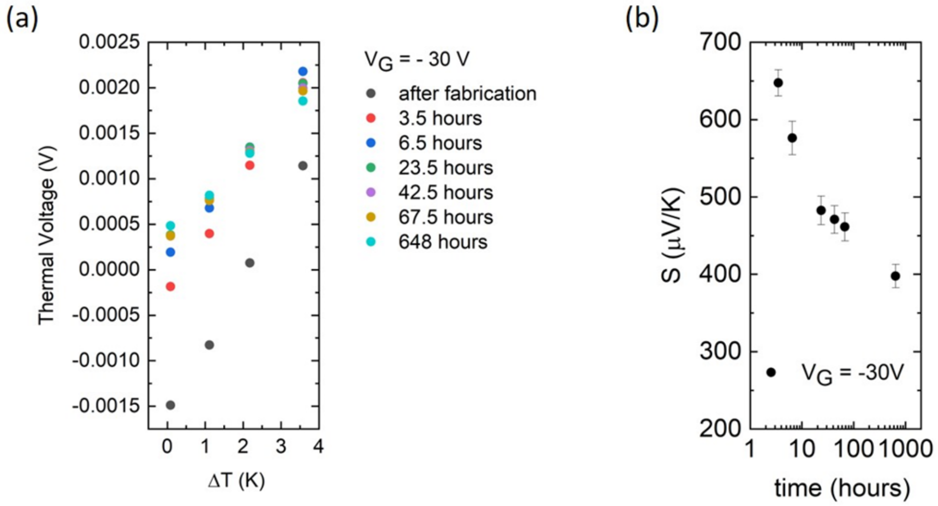

Supplementary Figure 5 Extraction of the gate modulated Seebeck coefficient in the organic semiconductor. (a) thermal voltage with applied temperature difference as a function of time in air for a given gate voltage, (b) extracted Seebeck coefficient plotted with time at this gate voltage.

SI Section 6: Spectral Analysis of the Density of Trap States

To quantify the dynamics of the trap states during device aging, we monitored the evolution in the trap density of states (t-DOS) as a function of time; results are shown in Supplementary Figure 6. These curves are derived from the transistor measurements (Fig. 2 (c) of the main paper) and provide information both on the trap densities and their energetic distribution (energy with respect to the valence band edge).

To extract the t-DOS, first the channel conductivity σ was evaluated as a function of gate voltage from the linear regime of the transfer characteristics for each panel shown in Fig. 2 (c) of the main paper using:

$$\sigma(U_{GS}) = \frac{L}{W}\frac{I_D}{V_{DS}} \quad (1)$$

where U_{GS} is the gate-source voltage above the flat-band voltage V_{FB} ($U_{GS}=|V_{GS}-V_{FB}|$) and V_{FB} was approximated to be equal to the turn-on voltage (V_{ON}). Bending of the energy level at the semiconductor/dielectric interface with increasing the gate-voltage introduces an interface potential $V_0(U_{GS})$ that was obtained by numerically solving:

$$exp\left(\frac{eV_0}{kT}\right) - \frac{eV_0}{kT} - 1 = \frac{e}{kT}\frac{\varepsilon_i d}{\varepsilon_s l \sigma_0}\left[U_{GS}\sigma(U_{GS}) - \int_0^{U_{GS}} \sigma\left(\widetilde{U_{GS}}\right) d\,\widetilde{U_{GS}}\right] \quad (2)$$

where ε_i and l represent the dielectric permittivity and thickness, respectively, ε_s and d are the polymer semiconductor permittivity and the thickness, σ_0 is the conductivity at flat band voltage. Next, the total hole density $p(V_0)$ was calculated from:

$$p(V_0) = \frac{\varepsilon_0 \varepsilon_i^2}{\varepsilon_s l^2 e} U_{GS}\left(\frac{dV_0}{dU_{GS}}\right)^{-1} \quad (3)$$

The trap DOS was evaluated by numerically differentiating $p(V_0)$ with respect to V_0:

$$N(E) \approx \frac{1}{e}\frac{dp(V_0)}{dV_0} \quad (4)$$

Here E represents the energy of the trapping state relative to the Fermi level.

In Supplementary Figure 6 (a), we include a representative curve from each state of the device, while in Supplementary Figure 6 (b) we include the complete set of curves along with their corresponding times (offset for clarity). The t-DOS is following an exponential distribution typical for polycrystalline and amorphous organic semiconductors, where the electronic states tailing into the bandgap are the result of structural defects and/or

impurities present in the film. The peak found in the curves immediately after fabrication confirms that discrete electronic states are present in the bandgap. These states are eliminated upon extended exposure to ambient air, as evidenced by the fact that the peak is gradually suppressed. Additionally, the increase in the density of states very close to the top of the valence band (shallow traps) supports the hypothesis that oxygen doping occurs during the first several hours of exposure, *i.e.*, in the first regime (initial 10 hours). Later, the overall density of trap states is reduced, most likely due to microstructural changes taking place in the film upon solvent removal, as discussed in detail in the main manuscript.

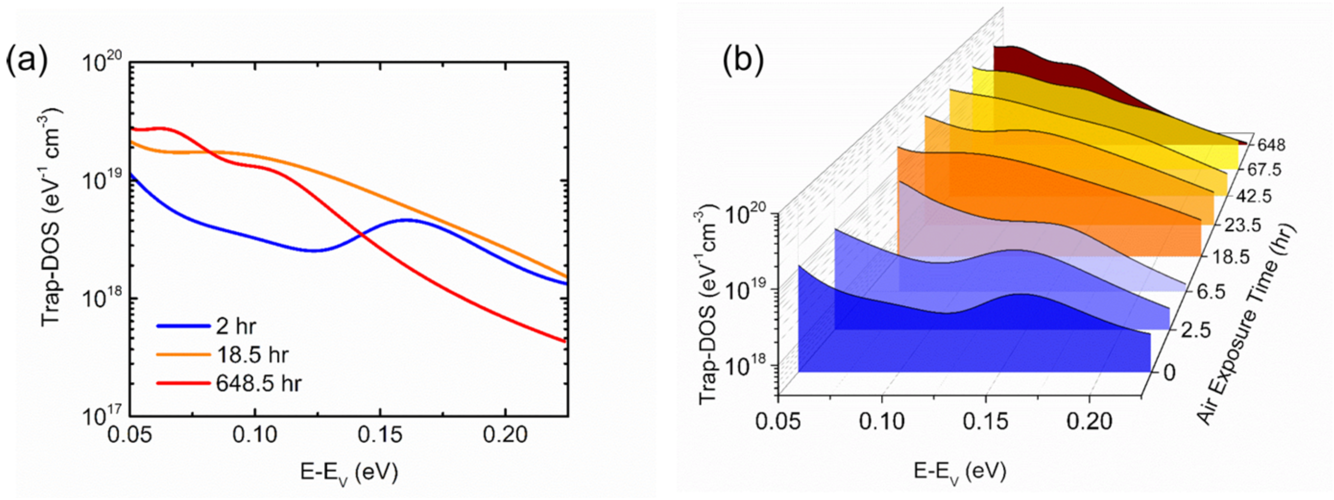

Supplementary Figure 6 Trap DOS spectra evaluated at different times during device operation. (a) A subset of 3 curves, each corresponding to one state of the device and highlighting the dynamic processes occurring in the film. (b) Complete dataset offset for clarity. All curves are plotted as a function of energy from the valence band edge.

SI Section 7: Molecular Mechanics and Molecular Dynamics Numerical Simulations

Methodology

To get structural insights on the impact of oxygen molecules in films of IDTBT, Molecular Mechanics (MM) and Molecular Dynamics (MD) simulations have been performed on two systems containing 20 IDTBT hexamers (System I) and 20 IDTBT hexamers and 120 O_2 molecules (System II). In each case, the supramolecular organization of the films have been studied for the amorphous phase following a protocol adapted from our previous work [V. Lemaur *et al.*, Chem. Mater. 2019]. Briefly, the hexamers and the oxygens are first randomly distributed in a large unit cell (300 Å x 300 Å x 300 Å) that is replicated using periodic boundary conditions to build the 3D solid. All MM/MD calculations have been performed with the Materials Studio (MS) 2018 package using a force-field derived from Dreiding where the torsion potentials between adjacent subunits and between the conjugated cores and the alkyl chains have been reparametrized against Density Functional Theory (DFT) calculations (at the B3LYP/cc-pvtz level of theory) performed using Gaussian09 suite. The atomic charges have been obtained by fitting the electrostatic potential (ESP charges) on an isolated dimer, calculated at the same level of theory. The electrostatic term follows Coulomb's law (r^{-1}) instead of the potential in r^{-2} defined by default and is calculated using the Ewald summation method. The van der Waals parameter associated to the hydrogen atom has been set to 2.50 Å instead of the 3.195 Å default value since recent studies showed its inadequacy [S. Hoyas, V. Lemaur, Q. Duez, F. Saintmont, E. Halin, J. De Winter, P. Gerbaux, J. Cornil, Adv. Theory Simul. 2018, 1800089; S. Saiev, L. Bonnaud, P. Dubois, D. Beljonne, R. Lazzaroni, Polym. Chem. 2017, 8, 5988; A.R. Kulkami; D.S. Sholl, Langmuir 2015, 31, 8453]. Once the unit cell is built, it is subjected to a 500 ps MD run at high temperature (NVT; T= 1000K) while keeping the density low (~0.02 g/cm^3) to favor a random spatial distribution of the oligomers. Then, five successive 500 ps-long MD runs (NPT, P = 1atm) were performed at decreasing temperature (1000K, 500K, 400K, 350K, 298K) and, finally, a 2 ns-long MD simulation (NPT; P = 1 atm, T = 298K) is performed and snapshots are saved every 5 ps for further analysis.

To characterize the distance between the chains (or their subunits) and the oxygens, the radial distribution functions between the different reference points have been built from the 401 snapshots of the 2 ns-long MD trajectory, taking as reference points different regions of the donor and/or acceptor, see Supplementary Figure 7.

The torsion angle distribution profiles have been built from all torsions among all chains in the 401 snapshots and all the torsions ranging from 180° to 360° have been converted into the 0°-180° range.

Results

The radial distribution functions between the oxygens and the different parts of the IDTBT unit are showed in Supplementary Figure 7 for System II. The interactions are quite similar for all pairs of atoms except that there is a slightly more intense curve for the interactions with the sulfur atom of the BT unit and a shift to smaller distances for the nitrogen atoms. These results suggest that oxygens are distributed everywhere within the films with a slight excess of probability close to the BT unit.

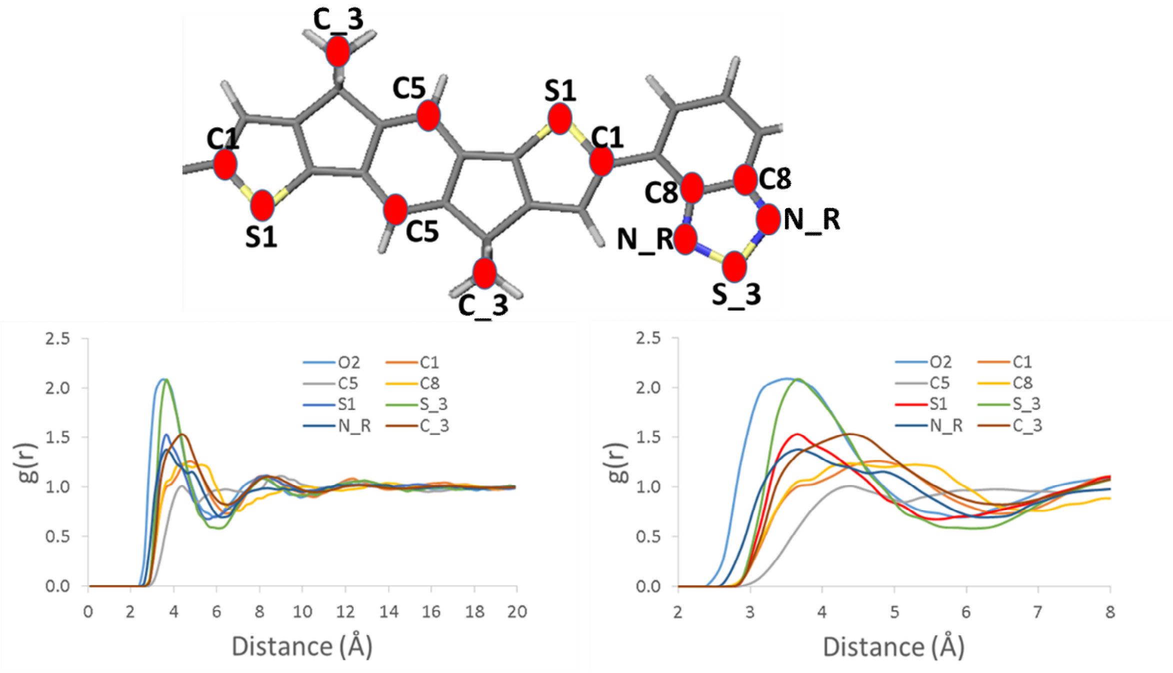

Supplementary Figure 7 Radial distribution functions between the oxygen molecules and different parts of IDTBT (labelled in the upper panel) in System II, as averaged over 401 morphologies extracted from the 2ns-long MD

As seen in Supplementary Figure 8, the analysis of the torsion distribution profiles of the amorphous phase shows that there is no difference between the systems in presence or absence of oxygens and that intramolecular charge transport will not be affected by fluctuations of torsions along the chains.

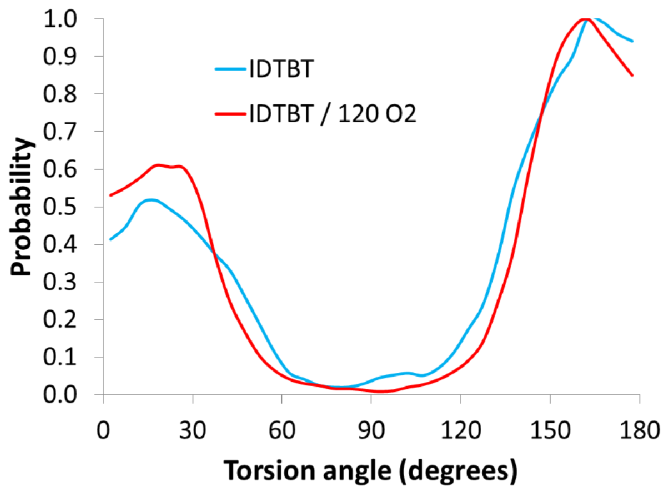

Supplementary Figure 8 Torsion distribution profiles of IDTBT films containing oxygen molecules (in red) or without oxygen molecules (in blue) averaged over all the inter-ring torsion along the polymer chains for the 401 morphologies extracted from 2ns-long MD analysis.

SI Section 8: A note on the slopes of the Seebeck coefficient with carrier density

When plotting the slope of the Seebeck coefficient with carrier density, several interpretations exist on how the carrier density must be computed within the channel of the device. The work of Batlogg and Pernstich (Nature Materials 2008) as well as the work of Kemerink and Germs (Physical Review Letters 2012) shifted the applied gate voltage by the transistor threshold voltage before computing the carrier density for the Seebeck coefficient vs carrier density plot.

This scaling of the gate voltage by the threshold voltage in the voltage-modulated Seebeck does not consider that a large lateral/in-plane electric field is not under application between the source and drain electrodes when the thermal voltage for the Seebeck coefficient is being measured. For this reason, the work of Venkateshvaran and Nikolka (Nature 2014) used a voltage scaling not from the transistor threshold voltage, but by looking at the onset voltage from the capacitance-voltage plot within the device. This is because the capacitance-voltage is closest to the conditions represented in the gated Seebeck measurements, i.e., there is no applied lateral source-drain voltage in either measurement.

When measuring the Seebeck coefficient in several ambipolar organic semiconductors done by Broch and Venkateshvaran (Advanced Electronic Materials 2017), a dilemma arose as to what scaling voltage needs to be chosen seeing as a third voltage, i.e., the so-called turn-on voltage (namely the point in the ambipolar transfer characteristics where the current shifts from being dominantly hole to dominantly electron) can also be used. Consistency was however maintained in the case of the ambipolar Seebeck measurements to draw comparative conclusions that were robust.

In the current work, we have chosen to plot the Seebeck coefficient vs the carrier density in the main manuscript by not shifting or scaling the carrier density unnecessarily. This is because the device was seen to turn on at close to 0 gate voltage bias from the transistor characteristics for holes, and because the gate voltages applied are large, i.e., between -30 V and -50V, thus potentially larger than any small turn on voltage that might otherwise be factored into the measurements. The consequence of this straightforward plot of the Seebeck coefficient versus the carrier density is that the slope in figures such as Fig. 3 (h) of the main manuscript are upper bounds rather

than lower bounds. It means that the slopes of the Seebeck coefficient versus the carrier density would be lower than these numbers should any other scaling voltage be used for the carrier density computation with a negative gate voltage threshold, turn-on, or on-set. This was why our earlier work (Nature 2014) reported f = 0.3, while we report f = 0.37 in this current manuscript under the same conditions. The trapping fraction f = 0.37 shown in Fig. 3 (h) of the main manuscript represents an upper limit on the slopes of the Seebeck measurements carried out within one week of fabrication and after curing in air.

SI Section 9: Nanomechanics of C16-IDTBT samples

9.1 Effect of annealing time

The data presented in the main manuscript has suggested that the solvent content of C16-IDTBT sample is of paramount importance for the nanomechanical response of the thin film. As the solvent content in the film is reduced with annealing time, we compared one sample annealed for only 1 min at 100 °C with a sample annealed for the standard time of 1 hour at the same temperature. The data in terms of elastic modulus and adhesion maps are provided in Supplementary Figure 9 and are obtained using a JPK NanoWizard 3 Atomic Force Microscope (JPK Instruments AG, Berlin, Germany) in the Quantitative Imaging (QI) mode with high-speed data capturing. Tap300DLC probes (Budget Sensors) were used. The nominal spring constant of the probes is 40 N/m as calibrated prior to the measurements. The determined probe tip radii were 10-12 nm and the set point was 100 nN in all measurements.

The sample annealed for only 1 min contains such a large amount of solvent that it is significantly softer than the sample annealed for 1 hour. The data also demonstrates the presence of trapped voids or drops in the film, seen as dark round areas in the modulus image. Without chemical analysis it cannot be determined if these features are due to remaining solvent droplets or due to voids left by solvent droplets that have been evaporated. However, it is clear that the C16-IDTBT thin film is less homogeneous and less well packed at the shorter annealing time, which explains the lower modulus. The presence of the larger voids/drops can also be seen in the adhesion image. Note that the scale of these images are 10 x 10 μm^2, and hence no nanoscale features can be observed.

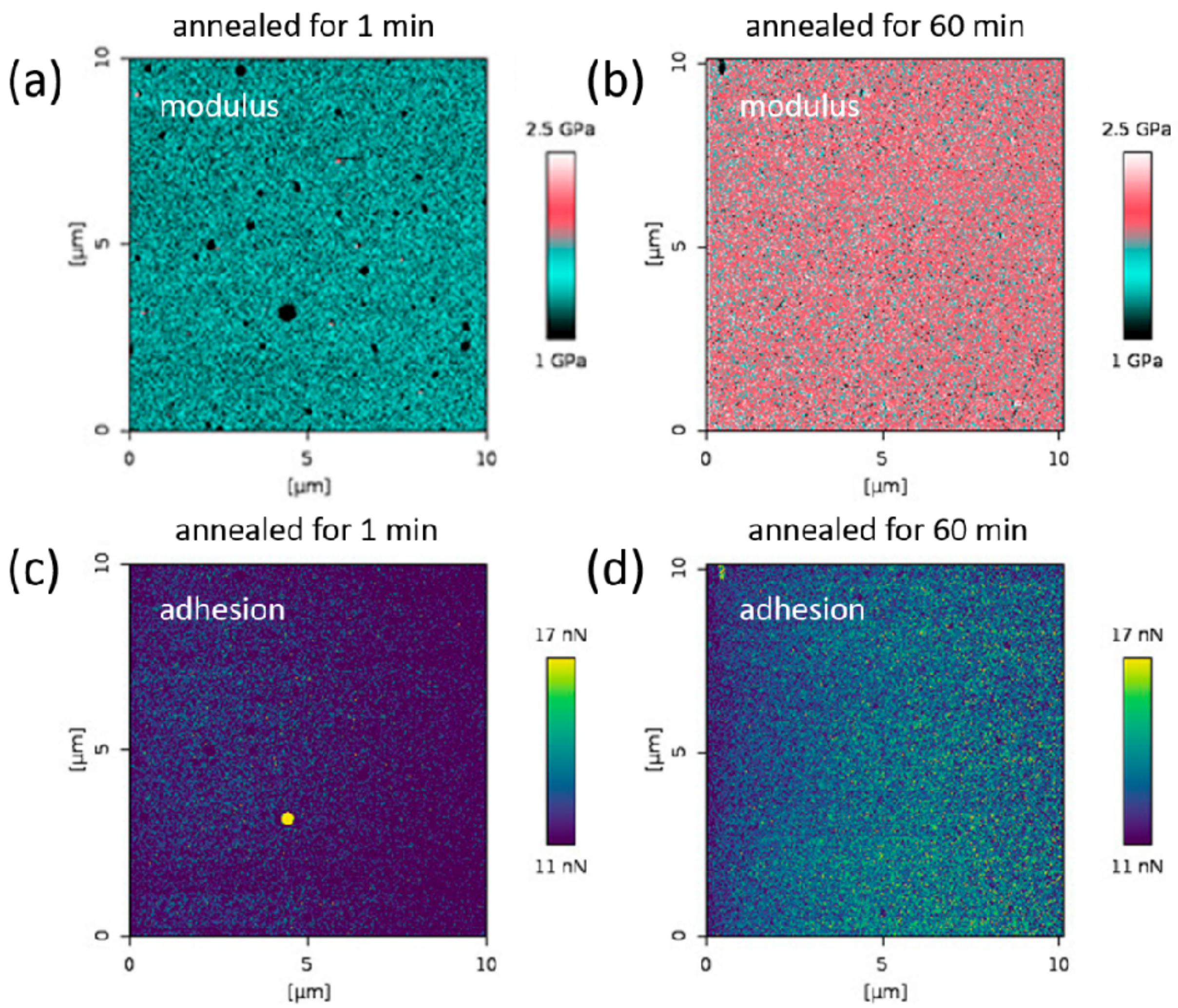

Supplementary Figure 9 The evaluated nanomechanical property maps, Modulus and Adhesion, for a sample annealed at 100 °C for 1 min [(a) and (c)], and for 60 min [(b) and (d)] after spin coating. The samples were stored in nitrogen for several days and measured about 1 hour after opening. The elastic modulus maps are shown in the top row and adhesion maps in the bottom row.

9.2 Viscoelasticity and force curve hysteresis

The elastic modulus is an important materials characteristic and it can be evaluated from AFM nanomechanical measurements. However, one needs to be critical as in this manuscript it is evaluated by fitting an elastic contact mechanics model to the measured force curves, and this does not consider viscoelastic effects. It is thus important to consider the force curves that are utilized for constructing the nanomechanical maps. Typical force-distance curves for the two annealing times are shown in Supplementary Figure 10. We note that the forces measured on

approach are slightly larger than measured on retract. Thus, there is a certain force distance hysteresis that signifies that the deformation is not purely elastic. The hysteresis is, however, small and we conclude that the modulus extracted for these films are reliable. The values we obtain are 1.30 ± 0.19 GPa and 1.94 ± 0.18 GPa for the sample annealed for 1 min and 1 hour, respectively.

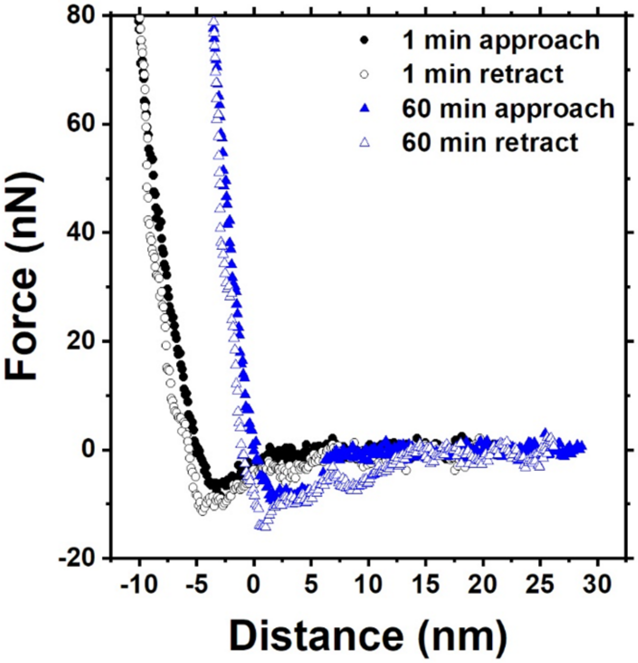

Supplementary Figure 10 Typical force curves measured on the samples annealed for 1 min and for 60 mins that were stored in nitrogen for several days prior to measurements. A difference in the approach and retract curves, i.e., in the short-range repulsion region, suggests a stiffer surface response for the sample annealed for 60 min compared to the sample annealed for 1 min. In addition, the force curves measured on retraction indicate polymer bridging as the attraction persists to 15 nm and the attractive part of the force curve has a non-smooth nature, which is a sign of bridging [See Thormann, E., Simonsen, A. C., Hansen, P.L., and Mouritsen, O.G., Langmuir 24, 7278-7284 (2008)]. For clarity, an offset of -5 nm was added in the case of the sample annealed for 1 min.

9.3 Intermodulation AFM (ImAFM) measurements of elastic modulus

There are different AFM modes that can evaluate the elastic modulus also for viscoelastic materials. One such mode is Intermodulation AFM. ImAFM, rather than measuring force curves, utilizes intermodulation productions

of cantilever excitation frequencies to extract in-phase (elastic) and out-of-phase (viscous) contributions to tip-surface interactions. [For details we refer to work of Haviland, e.g. Haviland, D.B., van Eysden, C.A., Forchheimer, D., Platz, D., Kassa, H.G., and Leclére, P. Soft Matter 12, 619-624 (2016)]. The Amplitude Dependent Force Spectroscopy (ADFS) method is further used to reconstruct the nonlinear tip-surface force and create a force volume data set for nanomechanical evaluation [For details we refer to the work of Forchheimer D., Platz D., Tholén E., and Haviland D., Phys. Rev. B 85, 195449, (2012)]. To check if the modulus reported above agrees with the modulus obtained by ImAFM, we also investigated stabilized C16-IDTBT films with ImAFM and the data are provided in Supplementary Figure 11.

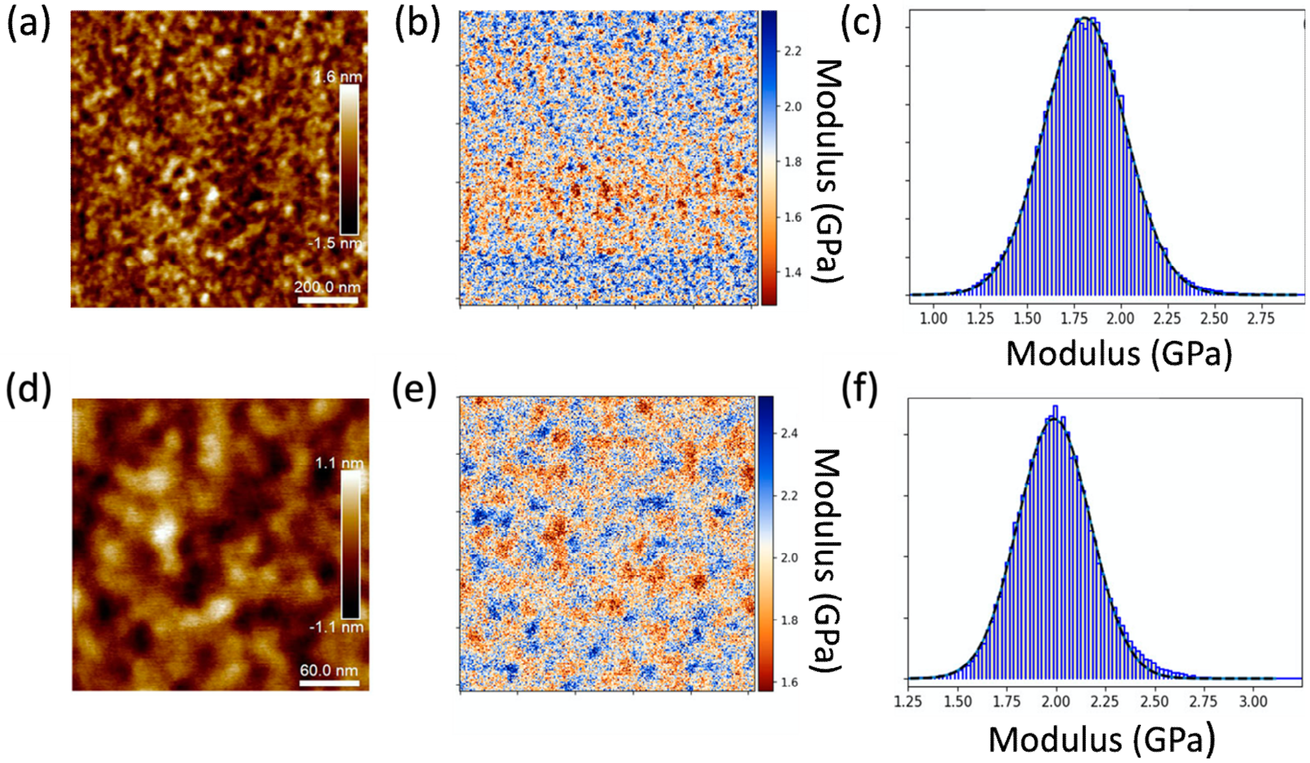

Supplementary Figure 11 Topography [(a) and (d)], Modulus maps [(b) and (e)], and corresponding modulus histograms [(c) and (f)], obtained by ImAFM on stabilised C16-IDTBT films. Top row (a) and (b) has an image size 1x1 μm^2. Bottom row (d) and (e) has an image size 0.3 x 0.3 μm^2. A Gaussian function was fit to the histograms to evaluate the average modulus value.

Indeed, also with ImAFM we obtain an average modulus of around 2 GPa, which confirms that the small hysteresis observed in Supplementary Figure 10 does not invalidate the modulus measurements with the force curve-based

techniques. In the 0.3 x 0.3 μm^2 image, we once again also clearly see the harder and softer regions discussed in the main manuscript as arising due to differences between ordered and disordered regions.

SI Section 10: Time evolution of a C16-IDTBT sample with plasticizer contaminants

In this supporting section, we measure the nanomechanical properties of C16-IDTBT films that are artificially and deliberately contaminated with softer nanoscale plasticizers that have a very different modulus to C16-IDTBT. The average apparent modulus values measured using AFM based nanomechanical measurements on fresh C16-IDTBT thin films that were not left to age more than a week are shown in Supplementary Figure 12 (a, b, c, d). The modulus of the C16-IDTBT regions can be artificially high because of sample viscoelasticity, since the samples were measured relatively soon after fabrication as shown in Supplementary Figure 12. If the same plasticizer samples are left to sweat out the residual solvent from the film over time, the viscous response becomes less important, and the modulus arrives at its stabilised limit of 2 GPa that we showed in this current work. The line scan across the plasticizer in Supplementary Figure 12 shows that the modulus of the plasticizer is only marginally reduced over time. This is because the plasticizer is assumed to be saturated with small amounts of C16-IDTBT and the C16-IDTBT is assumed to be saturated with small amounts of plasticizer in these films. The modulus of the plasticizer nevertheless undergoes only a small change with time compared with the surrounding C16-IDTBT. This is because the plasticizer does not sweat out as much solvent as the surrounding IDTBT polymer film.

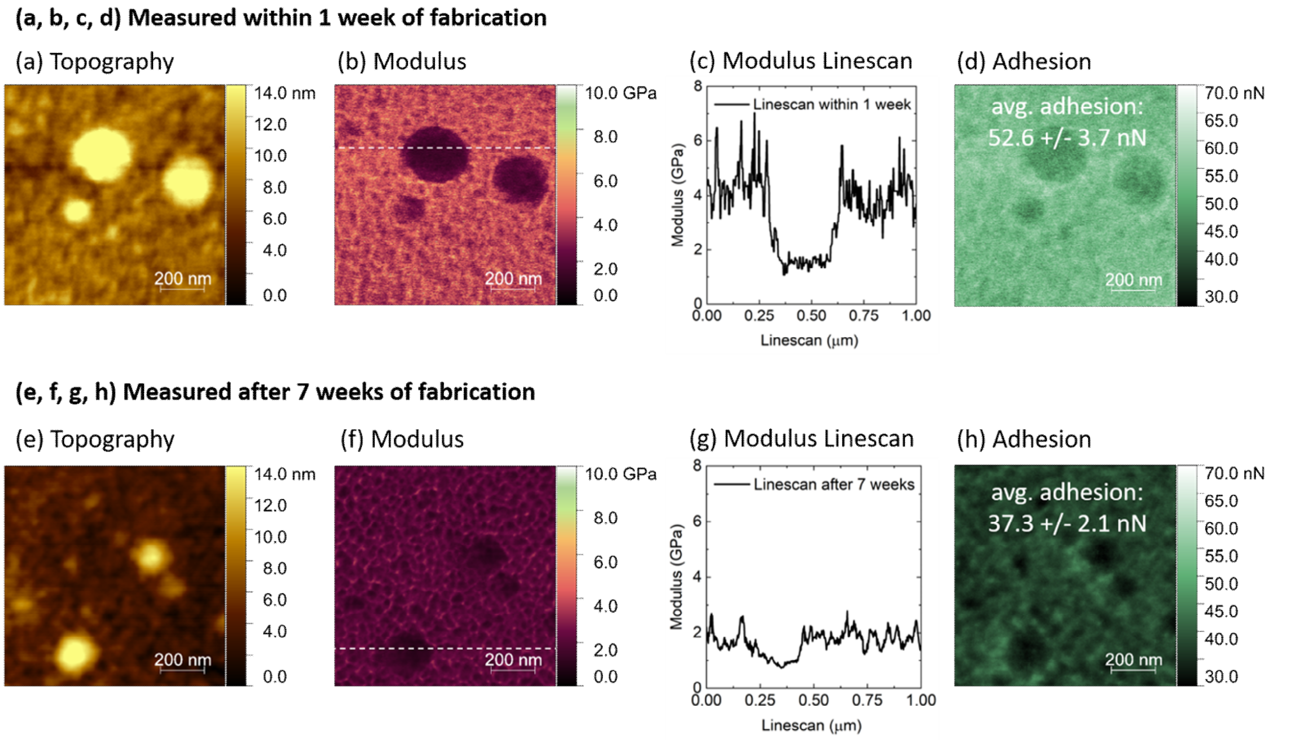

Supplementary Figure 12 (a), (b), (c), (d) are the topography, modulus, modulus line scan and adhesion in a C16-IDTBT sample containing an organic plasticizer and measured within the first week from device fabrication. (e), (f), (g), (h) are the topography, modulus, modulus line scan and adhesion in a C16-IDTBT sample containing an organic plasticizer and measured after seven weeks from device fabrication. In the latter, the C16-IDTBT was allowed to sweat out its residual solvent over time, and so its modulus reduced in addition to its surface adhesion.
